# Use of inhaled ciclesonide for treatment of moderate asthma in Thoroughbred racehorses

**DOI:** 10.1111/jvim.17267

**Published:** 2025-02-13

**Authors:** Macarena G. Sanz, Georgia Jellen, Lauren Cody, Jenyka Bergsma, Mandy Cha, Clark Kogan, Gordon Kordas, Warwick M. Bayly, Renaud Leguillette

**Affiliations:** ^1^ Washington State University, College of Veterinary Sciences Pullman Washington USA; ^2^ Faculty of Veterinary Sciences University of Calgary Calgary Alberta Canada; ^3^ Cavalli Equine Veterinary Services Auburn Washington USA; ^4^ Simkins and Tingdale, Inc. Auburn Washington USA; ^5^ StatsCraft LLC Spokane, Washington 99203 USA; ^6^ Statistical Consultant Reno Nevada USA

**Keywords:** Aservo EquiHaler, bronchoalveolar lavage, corticosteroids, cytokines, endoscopy, mastocytes, tracheal mucus

## Abstract

**Background:**

Mild‐moderate asthma is common in horses. Inhaled ciclesonide has been approved only for treatment of severe asthma in horses.

**Hypothesis/Objectives:**

We hypothesized that a 10‐day treatment course of inhaled ciclesonide (Aservo EquiHaler) would improve clinical signs, endoscopic tracheal mucus scores, and bronchoalveolar lavage fluid (BALF) cytology in racehorses with moderate asthma.

**Animals:**

Racehorses with moderate asthma housed at the Emerald Downs Racetrack in Auburn, WA.

**Methods:**

Prospective, randomized, double‐blinded, placebo‐controlled clinical study. Horses received inhaled ciclesonide (n = 12) or placebo (n = 9) for 10 days. Clinical signs were assessed at different times using the Tesarowski and HOARSI scores and cough during exercise. Endoscopy scoring, BALF cytology, and BALF cell selected gene expression (RT‐qPCR) were assessed on Days 0 and 10. Linear and ordinal simple and mixed effects models were used to analyze the data using R statistical software. Significance was set at *P* < .05.

**Results:**

Only treated horses showed a decrease over time in HOARSI (*P* ≤ .001), cough (*P* = .001; −2.83 [−4.37, −1.29]), mast cell % (*P* = .03; −2.68 [−4.36, −1.0]) and relative expression of IL‐6 (*P* = .002; 0.44 [0.04, −6.99]) and IL‐13 (*P* = .03; 0.52 [0.04, −7.89]) in BAL cells. Treated horses had lower HOARSI (*P* = .002; −1 [1, 1]) and mast cell % (*P* = .02; −2.96 [−5.52, −0.39]) on Day 10.

**Conclusion and Clinical Importance:**

Treatment with inhaled ciclesonide improved clinical signs and decreased BALF mastocytic inflammation in racehorses with moderate asthma without change in the environment. Treatment effect on neutrophilic or eosinophilic asthma remains undetermined. The small number of horses was a study limitation.

**Abbreviations**
BALF: bronchoalveolar lavage fluid
GAPDH: glyceraldehyde 3‐phosphate dehydrogenase
HOARSI: Horse Owner Assessed Respiratory Signs Index
HPRT1: hypoxanthine phosphoribosyltransferase 1
ICAM: intercellular adhesion molecule 1
IFNγ: interferon‐γ
IL‐10: interleukin 10
IL12A: interleukin 12A
IL‐12B: interleukin 12B
IL‐13: interleukin 13
IL‐17A: interleukin 17A
IL‐1β: interleukin IL‐1β
IL‐23A: interleukin 23A
IL‐4: interleukin 4
IL‐6: interleukin 6
IL‐8: interleukin 8
REST: relative expression software tool
Th17: T helper 17 cells
TNFα: tumor necrosis factor‐α
β‐Gus: β‐glucuronidase

## 1 INTRODUCTION

Mild to moderate asthma in horses is defined as a combination of respiratory clinical signs and lung inflammation. The term mild asthma is used when horses have lower airway inflammation identified by abnormal bronchoalveolar lavage fluid (BALF) cytology or abnormal lung function, with presenting clinical signs that might be limited to decreased performance.^1^ Moderate asthma refers to horses that have lower airway inflammation combined with respiratory clinical signs such as cough, increased respiratory rate during work or the post‐exercise recovery phase but do not have labored breathing at rest.^2^, ^3^, ^4^ Mild to moderate equine asthma has a higher prevalence than severe asthma in horses,^5^ is common in Thoroughbred racehorses, and reduces performance and aerobic capacity.^6^, ^7^ Definitive diagnosis is based on an abnormal percentage of neutrophils (>5%), mast cells (>2%) or eosinophils (1%) in the BALF of horses with compatible clinical signs.^1^, ^3^, ^6^

Treatment of mild to moderate asthma typically involves a combination of environmental management, which is difficult to achieve in racing barns, and administration of corticosteroids. Inhaled corticosteroid therapy delivers the medication directly into the lower airways and fluticasone therapy is beneficial in treating mild/moderate asthma.^8^ Dexamethasone, fluticasone and beclomethasone by inhalation are used off label for the treatment of asthma, but can cause systemic cortisol suppression.^9^, ^10^, ^11^ Moreover, inhaled dexamethasone might result in increased expression of proinflammatory genes in the lower airway.^12^ Ciclesonide is an inhaled corticosteroid prodrug with a low affinity for glucocorticoid receptors when in the inactive form.^13^ The prodrug is de‐esterified in the lung to the active form desisobutyryl‐ciclesonide (des‐CIC) and does not affect serum cortisol concentration, thus making it a safe therapeutic choice for horses with asthma.^13^, ^14^, ^15^ In a large field study, a 10‐day treatment course of inhaled ciclesonide reduced clinical signs of severe asthma and was well tolerated by the majority of the horses.^16^ The ciclesonide inhaler available in North America (Aservo Equihaler, Boehringer Ingelheim Animal Health USA Inc., Duluth, Georgia, USA) has a short withdrawal period of 48 hours for racing. However, data regarding efficacy in mild to moderate asthma have not been reported.

We hypothesized that the manufacturer's recommended 10‐day treatment with inhaled ciclesonide would improve clinical signs of moderate asthma in racing horses. To test this hypothesis, we evaluated the effect that a 10‐day treatment course of inhaled ciclesonide (Aservo EquiHaler) had on clinical signs, endoscopic tracheal mucus scores and BALF cytology and selected gene expression in racehorses diagnosed with moderate asthma. Although production of the inhaler used in this study will be discontinued in North America, the data presented in this paper remains relevant to the treatment of moderate asthma in racing horses as alternative administration devices for inhaled treatment currently exist in the equine market.

## 2 MATERIALS AND METHODS

### 2.1 Study design

This prospective, randomized, double‐blinded, placebo‐controlled clinical study was conducted with client‐owned racehorses at the Emerald Downs Racetrack in Auburn, WA after approval by the Institutional Animal care and Use Committee at Washington State University. Horses were included after the owner or the trainer consent was obtained.

After inclusion criteria for moderate asthma were met (see description below), horses were randomly assigned to receive inhaled ciclesonide (treatment group) or a placebo (inhaler device that produced a mist of the carrier solution without ciclesonide). No changes were made to the management or environmental dust control during the study. Horses were treated by the research personnel who were blinded to the treatment vs placebo inhalers. Treatment was administered by the researchers for 10 days as recommended by the manufacturer. Tracheal endoscopic findings, bronchoalveolar lavage cytology and cytokine expression of BALF cells were evaluated before (Day 0) and at the end of treatment (Day 10 ± 1). Clinical signs of respiratory disease were assessed throughout the study (see description below).^17^

### 2.2 Inclusion criteria

Thoroughbred horses in training or racing that had a cough or clear nasal discharge at rest or during exercise, had a post‐exercise tracheal endoscopic mucus score ≥2/5 (moderate larger blobs)^18^ and a bronchoalveolar lavage cytology consistent with mild‐moderate asthma (>5% non‐degenerative neutrophils, >2% mast cells or >1% eosinophils) were included in the study.^3^, ^6^ For a week before, and while in the study, horses did not receive any other treatments. Horses with no clinical signs such as cough or nasal discharge but with BALF abnormalities (subclinical asthma) were not included.^1^, ^19^

### 2.3 Respiratory clinical signs

Horses' clinical signs of respiratory disease were evaluated using 3 scoring methods: A clinical exam developed by Tesarowski et al. using a modification of a previously published system,^20^ referred to as the Tesarowski clinical scoring system for the purposes of this study, a modified HOARSI, and the number of coughs during exercise.^2^, ^18^, ^21^, ^22^ The Tesarowski score was performed on Days 0 and 10 by 2 individuals who were blinded to the horse's treatments. Horses were clinically examined and given a weighted score (Table 1) using variables previously described with the addition of a rebreathing examination.^20^ The total score for each horse was used for the analysis.

**TABLE 1:** Tesarowski weighed clinical scoring system with the addition of a rebreathing examination.^19^

Variable	Description	Score
Respiratory rate (breaths/min)	<16	0
	16‐20	1
	21‐25	2
	26‐30	3
	>30	4
Nasal discharge presence	None	0
	Serous	1
	Mucopurulent	2
Nasal discharge amount	None	0
	Small amount: Fill of nostril <1/3 ± upper lip 1‐2 thin streams	1
	Medium amount: Fill of nostril 1/3 to 2/3 ± upper lip 1‐2 thin streams	2
	Significant amount: Fill of nostril >1/3 ± upper lip >2 finger wide stream	3
Abdominal lift	None	0
	Mild (perceptible heave line)	1
	Pronounced (abdomen, thorax, and anal movement)	2
Nasal flaring	None	0
	Present	1
Tracheal sounds (rattles)	Normal (tubular sound)	0
	Increase in intensity	1
	Mucus movement	2
Bronchial tones	Normal (may hear BV sounds cranio‐ventrally)	0
	Audible ventral and dorsal sounds (BV lung sounds everywhere without a bag)	2
Crackles	None	0
	Present	2
Cough during exam	None	0
	Inducible by tracheal massage	1
	Intermittent	2
	Paroxysmal/frequent	3
Using a rebreathing bag Crackles	None	0
	Present	2
Using a rebreathing bag Wheezes	None	0
	Present	2
Total maximum		30

*Note*: Horses were assessed on Days 0 and 10 and their total score each day was used for data analysis.

The HOARSI scoring, which is based on owner‐reported clinical history, has been used to assess horses with severe asthma.^2^, ^21^, ^22^, ^23^, ^24^ For the purposes of this study, an abbreviated version of this scoring system was used (Table 2). The information provided by grooms and trainers was recorded every morning, from Day 0 to Day 10 of the study to create individual daily HOARSI scores.

**TABLE 2:** Abbreviated Horse Owner Assessed Respiratory Signs Index (HOARSI) scoring system used daily to evaluate respiratory clinical signs in the stall.

Clinical signs description	Score
No episodes of coughing or nasal discharge	1
Mucus nasal discharge, occasional cough, or both	2
Abnormal breathing, regular or frequent coughing, or both	3
Abnormal breathing, regular or frequent coughing accompanied by poor performance, or both	4

The number of coughs heard by the jockey during exercise was recorded. To this extent, evaluators asked the jockeys for the total number of coughs within 5 minutes of finishing the exercise with each horse.

### 2.4 Endoscopy

Tracheal endoscopy, including visualization of the carina, was performed in all horses. Endoscopies were performed without sedation 30‐90 minutes after exercise (walker or track‐training) prior to treatment (Day 0) and on Day 10 (+1) after inhalation of the ciclesonide or placebo. Videos of the endoscopy were recorded the appearance and volume of tracheal mucus was graded using a published scoring rubric score by consensus of 2 evaluators blinded to the treatment group.^25^, ^26^ Briefly, a score of 0 denoted no mucus present in the pharynx, larynx, or trachea. A score of 1 was given to horses with little mucus, visible as small blobs within the trachea. A score of 2 denoted moderate mucus in the trachea, as characterized by more frequent or moderately sized blobs. Confluent, stream‐forming mucus was scored as a 3, formed pools of mucus were scored as a 4, and profuse amounts of mucus were assigned a score of 5.^18^, ^27^ Horses were included in the study if the tracheal mucus endoscopic score was ≥2 on Day 0.

### 2.5 Bronchoalveolar lavage fluid collection

Bronchoalveolar lavage fluid collection was performed on Day 0 and Day 10 after treatment as described.^12^ Briefly, horses were sedated with a combination of xylazine (Rompun, Dechra Pharmaceuticals PLC, KS, USA, 0.3 to 0.4 mg/kg, IV) and butorphanol (Torbugesic, Zoetis Services LLC, New Jersey, USA, 20 to 30 μg/kg, IV). A specialized tube (3 m long, 10 mm diameter, MILA International Inc, Kentucky, USA) and routine technique were used for this procedure.^28^ During the passage of the tube down the trachea, several small boluses of a 0.5% solution of lidocaine hydrochloride (Xylocard, Astra Pharma Inc, Mississauga, Ontario, Canada) were administered (up to a maximum of 120 mL) to desensitize the airway mucosa. Once the tube was wedged in the bronchus, the tube's balloon was inflated and two, 250 mL boluses of sterile isotonic saline (0.9% NaCl) were rapidly instilled into the bronchus and aspirated through the tube using syringe suction. The last 120 mL of BALF was collected in 50 mL sterile conical tubes and was kept on ice until analysis.

### 2.6 Cytology

Cytology slides were prepared within 3 hours of fluid collection using a cytocentrifuge (100*g* for 4 minutes) and an automatic stainer (Hema‐Tek 2000 Bayer) with a Modified Wright Giemsa stain for better visualization of mast cells. A differential cell count was performed on 200 nucleated cells by an experienced individual (RL) who was blinded to treatment groups. Epithelial cells were not included in the differential count. Horses were included if the cytology on Day 0 was consistent with the diagnosis of mild‐moderate asthma as described above.^3^

### 2.7 Messenger RNA expression analysis of BALF cells

All the fluid collected from each BALF sample was transferred into 50 mL conical tubes and the surfactant was disposed of. The BALF was centrifuged (500*g* for 10 minutes) and all the cell pellets were combined into a tube for RNA preservation (Tempus blood RNA tube, Thermo Fisher Scientific Inc, Massachusetts, USA). Tubes were stored at −20°C until analysis. All Tempus blood RNA tubes were processed for total RNA extraction at the Gluck Equine Research Center, in Lexington, Kentucky as previously described.^29^ Briefly, RNA was isolated using a KingFisher Flex (Thermo Fisher Scientific Inc, Massachusetts, USA) and the MagMax Core kit (Applied Biosystems, California, USA) per the manufacturer's recommendations, except there was no DNase step and the pelleted RNA was resuspended in 600 μL viral lysis buffer (Invitrogen Corporation, California, USA).

Expression of the following genes was calculated using β‐glucuronidase (β‐Gus), glyceraldehyde 3‐phosphate dehydrogenase (GAPDH), hypoxanthine phosphoribosyltransferase 1 (HPRT1) and Intercellular Adhesion Molecule 1 (ICAM1) as reference genes for all the samples using the relative expression software tool (REST) as described.^12^, ^30^ The use of REST allowed for correction for PCR efficiency and normalization using multiple reference genes.^12^ Housekeeping genes were selected based on the stability of their expression.^31^ Commercially available primers and probes were used (Thermo Fisher Scientific): β glucuronidase (β‐GUS, Ec03470630_m1), glyceraldehyde 3‐phosphate dehydrogenase (GAPDH, Ec03210916_gH), hypoxanthine phosphoribosyltransferase 1 (HPRT1, Ec03470217_m1), Intercellular Adhesion Molecule 1 (ICAM1, Ec07080735_m1), Interferon‐γ (IFNγ, Ec03468606_m1), Interleukins (IL), IL‐1β (Ec04260298_s1), IL‐4 (Ec03468864_m1), IL‐6 (Ec03468678_m1), IL‐8 (Ec03468860_m1), IL‐10 (Ec03468647_m1), IL‐12A (Ec03468747_m1), IL‐12B (Ec03468777_m1), IL‐13 (Ec03470543_m1), IL‐17A (Ec03470096_m1), IL‐23A (Ec03468800_m1), and tumor necrosis factor‐α (TNFα, Ec03467871_m1).

### 2.8 Treatments

Horses were randomly allocated into treatment (ciclesonide inhalation spray, Aservo EquiHaler) or placebo (inhaler canister with the excipient but without ciclesonide, provided by the same company). The canisters were coded and completely covered to prevent identification of the treatment group by the people administering the treatments, the veterinarians performing the endoscopies, the grooms, jockeys, and trainers. After randomized allocation, horses were treated according to the product label for 10 days: 8 actuations (2744 μg ciclesonide) twice a day for the first 5 days, followed by 12 actuations (4116 μg ciclesonide) once a day on Days 6‐10. The same protocol was followed using the non‐drug inhalers (control group). Treatments were performed at 6 am and 4 pm. Difficulties with the inhaler administration (any puffs not well synchronized with inhalation, horse head tossing) were recorded.

### 2.9 Statistical analysis

Normality was assessed using the Shapiro‐Wilk test and the Brown‐Forsythe test was used to assess variances. The number of coughs and HOARSI outcomes were measured once a day for the duration of the study and was analyzed over 10 repeated measurements. All other outcomes had 2 repeated measurements (baseline and follow‐up on Day 10). Ordinal mixed effects models with a random intercept for subject and a time by treatment interaction fixed effect were fit to each of the ordinal outcome measures (HOARSI, Endoscopic, and Tesarowski). This is akin to a Wilcoxon test but accurately captures the longitudinal nature of the data. The continuous outcomes were analyzed using linear regression models with an indicator for treatment. Before analysis, quantitative PCR data were logarithmically transformed to achieve normality where possible.^32^ All data analysis was completed with the R statistical software (v4.3.2, R Core Team 2023). Mixed effects models were fitted via the nlme package (Pinheiro, Bates, R Core Team 2023). Mixed effects, ordinal models were fitted using the ordinal package (Rune H. B. Christensen, 2023). Cytokines were assessed using the relative expression software tool (REST), which allows for correction for PCR efficiency and normalization with multiple reference genes, as described before.^12^ A significance level of *P* < .05 was used for all analysis.

## 3 RESULTS

A total of 21 Thoroughbred horses were included in the study. Of these, 12 received the treatment and 9 received the placebo. Twelve of these horses were geldings and 9 were mares. Age ranged between 2 and 8 (median 2) years of age. Age was not different between groups (*P* = .53). The Tesarowski score was not different over time by group and was not different between groups at any timepoint (Table 3). The HOARSI score decreased over time (*P* ≤ .001), but did not change in the placebo group (*P* = .60). At the end of the study (Day 10), the HOARSI score was lower (*P*; median [25th and 75th percentile]) (*P* = .002; −1 [1, 1]) in treated than in placebo horses (Table 3). Similarly, the number of coughs during exercise decreased over time (*P*; median [95% CI]) in the treated horses (*P* = .001; −2.83 [−4.37, −1.29]) but did not change over time in the placebo group (*P* = .22; −1.12 [−3.01, 0.76]). The number of coughs was not different between groups on Days 0 or 10 of the study (Table 4).

**TABLE 3:** Variable results, median (interquartile range [IQR], 25th percentile, 75th percentile), for the variables evaluated on Days 0 and 10 for horses that were assigned to the treatment (n = 12) or placebo (n = 9) groups.

Variables	Treatment d0	Treatment d10	Placebo d0	Placebo d10
	Median (IQR)	Median (IQR)	Median (IQR)	Median (IQR)
Tesarowski score	3 (1, 3.2)	3 (2, 3)	4 (3, 5)	3 (2, 5)
HOARSI score	2 (2, 3)	1 (1, 1)^#^ ^,^ *	2 (2, 2)	2 (2, 2)
Endoscopy score	3.5 (2.5, 3.6)	3 (1.8, 3.5)	3 (2.5, 3.5)	3 (2.5, 3)

Abbreviation: HOARSI, Horse Owner Assessed Respiratory Signs Index.

^#^ Significant differences from Day 0 values on each group.

* Significant differences between the groups at the specific time.

**TABLE 4:** Variable results, mean (95% confidence interval [CI]), for the variables evaluated on Days 0 and 10 for horses that were assigned to the treatment (n = 12) or placebo (n = 9) groups.

Variables	Treatment d0	Treatment d10	Placebo d0	Placebo d10
	Mean (CI)	Mean (CI)	Mean (CI)	Mean (CI)
Number of coughs	3.2 (2.0‐4.4)	0.4 (−0.7‐1.5)	1.7 (0.3‐3.7)	0.6 (−0.8‐2.0)
BALF mast cells (%)	4.6 (3‐6.3)	1.9 (0.3‐3.6)^#^ ^,^ *	5.5 (3.5‐7.4)	5.7 (3.8‐7.7)
BALF neutrophils (%)	6.3 (3.2‐9.5)	3.2 (0.05‐6.3)	7.3 (3.7‐10.9)	6.0 (2.3‐9.6)
BALF eosinophils (%)	0.5 (0.04‐1.1)	0.1 (−0.4‐0.6)	0.1 (−0.5‐0.7)	0.2 (−0.4‐0.8)

Abbreviation: BALF, bronchoalveolar lavage fluid.

^#^ Significant differences from Day 0 values on each group.

* Significant differences between the groups at the specific time.

Tracheal mucus scores did not change over time in treated or placebo horses and were not different between groups on Days 0 or 10 (Table 3). A Day 10 BALF collection could not be performed in one of the placebo horses because the horse's racing schedule that prevented the use of sedation. On Day 0, all but 1 horse had an abnormal percentage of mast cells (>2%), 11 horses had an abnormal percentage of neutrophils (>5%) and 1 had an abnormal percentage of eosinophils (>1%). The percentage of mast cells significantly decreased in the treated horses over time (*P* = .03; −2.68 [−4.36, −1.0]) but did not change in the placebo group (*P* = .76; 0.27 [−1.65, −2.21]). The percentage of mast cells was significantly lower in treated horses than in control horses 10 days after treatment (*P* = .02; −2.96 [−5.52, −0.39]). The percentage of neutrophils and eosinophils was not different between groups at any timepoint (Table 4).

**FIGURE 1:**
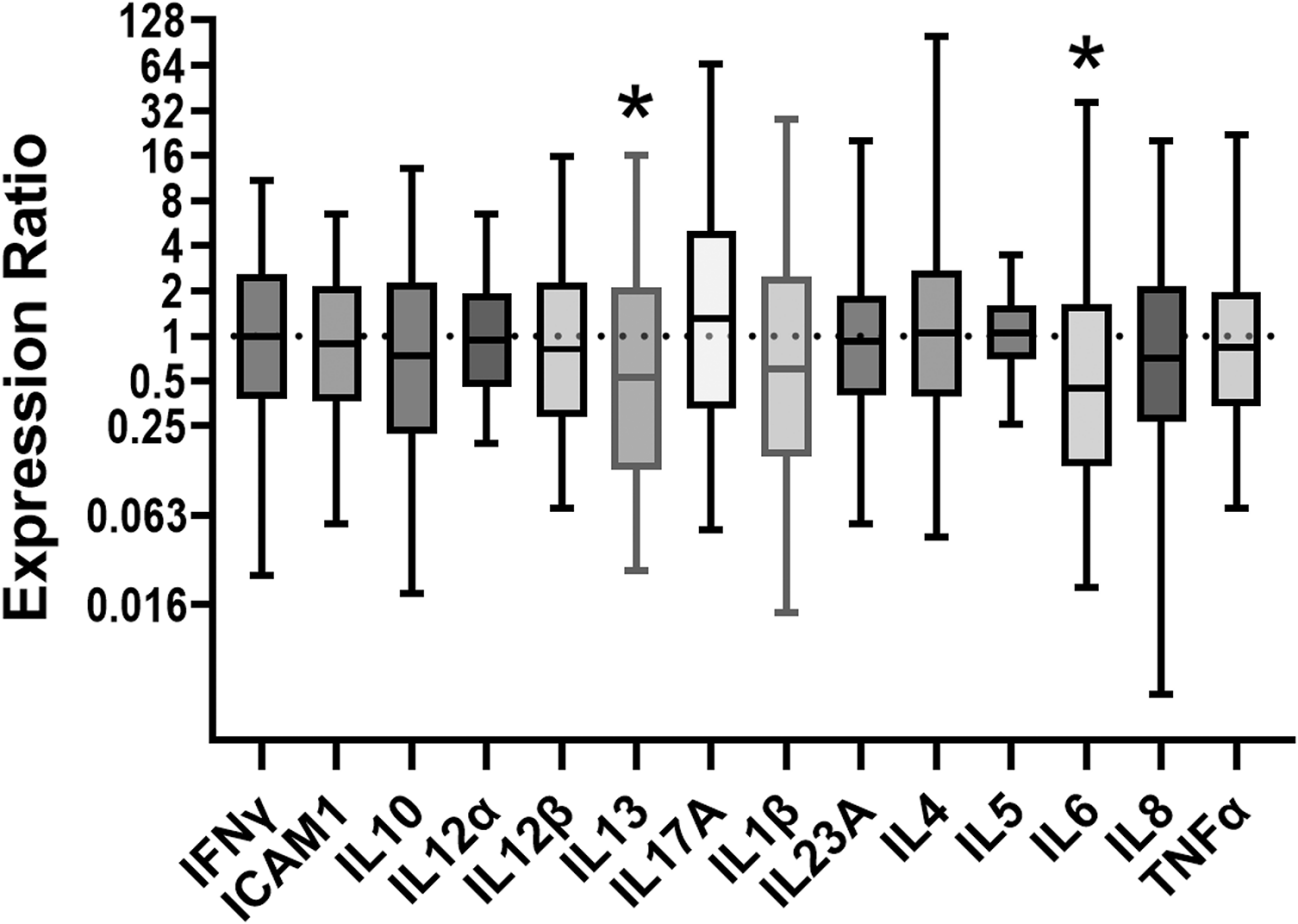
Bronchoalveolar lavage cell's relative cytokine expression of cytokines over time (Day 0 vs Day 10) in horses that received inhaled ciclesonide treatment (n = 12). An expression ratio of 1 (dotted line) indicates that there was no change in the relative expression of a particular gene over time. A ratio >1 indicates up‐regulation and <1 down‐regulation. The box represents the interquartile range (middle 50% of the data) and the whiskers indicate the minimum and maximum values. The line in the middle of the box represents the median. The asterisk (*) indicates that the change in the relative expression was significant (*P* < .05).

Cytokine expression of the BALF cells revealed no significant differences between groups at Days 0 or 10. There were no significant changes in cytokines overtime (d0 vs d10) in the placebo group. In the treated group, IL‐6 expression was downregulated by a mean factor of 0.44 (*P* = .002; 0.44 [0.04, −6.99]) and IL‐13 expression was downregulated by a mean factor of 0.52 (*P* = .03; 0.52 [0.04, −7.89]) (Figure 1).

## 4 DISCUSSION

Mild to moderate asthma, an environmentally induced, non‐septic inflammation of the lower airways, is common in racehorses.^6^ Our study shows that a 10‐day treatment with inhaled ciclesonide reduced the clinical signs and the BALF mastocytic inflammation in racing Thoroughbred horses with moderate asthma. Delivery of ciclesonide and placebo were both achieved using the Aservo EquiHaler system as previously reported,^16^ and the horses in this study tolerated the use of the device. The Aservo EquiHaler is a Soft Mist inhaler that produces a fine particle fraction that is deposited deep in the lungs.^16^ Production of this inhaler has been discontinued in 2024. Whether or not administration of ciclesonide with other commercially available nebulizers will achieve similar effects remains to be determined but based on the positive results observed in our study, this should be further investigated.

Ideally, pulmonary function testing for hyperresponsiveness of airways would have been performed to select horses for this study.^8^ Since these tests are not suitable for field studies, initial screening of horses was made based on clinical signs and the presence of mucus in the trachea. Horses were initially assessed using a combination of the Tesarowski, HOARSI clinical scores as previously described.^15^, ^16^, ^23^ These scores have been used in the past to predict pulmonary function in horses,^15^, ^16^ but can be insufficient to identify horses with milder clinical signs.^23^ The low Tesarowski score at the beginning of the study might explain the lack of discernable changes overtime. In contrast, the HOARSI score and number of coughs during exercise did change over time in treated horses only and the HOARSI score was lower in treated horses than in placebo horses after 10 days. The HOARSI score was developed for evaluation of asthma in horses and includes assessment of the respiratory pattern, cough, and nasal discharge.^22^, ^24^, ^33^ Cough is a sign of mild to moderate asthma, and it has been associated with neutrophilic inflammation.^34^ While cough is less common in young horses with asthma or in horses with mastocytic inflammation,^34^ occasional cough was not uncommon in the horses included in this study. Coughing during exercise and increased serous nasal discharge are specific but insensitive indicators of increased tracheal mucus.^35^ Since the horses in this study were only included if the tracheal mucus score was ≥2, this might explain the observed differences.

Visualization of mucus in the trachea has been correlated with inflammation and a tracheal mucus score of >2/5 in racehorses has been considered sufficient for the diagnosis of mild to moderate asthma.^3^ In this study, horses were included if they had clinical signs consistent with asthma and an endoscopic score of ≥2 as these have been shown to be a risk for poor performance in racehorses.^36^ An additional rationale for the use of endoscopy in our study was to rule out upper airway abnormalities that might have caused the clinical signs observed.^37^ The lack of changes in the amount of mucus in the trachea was not surprising as this has been observed in horses with severe asthma during disease remission.^23^ The study presented here was not designed to investigate the causes of this phenomenon, but long‐term hypersecretion of mucus and environmental stimulation have been proposed to contribute to the persistence of mucus in the trachea.^23^ Our study had sufficient power (80% with an alpha of 5%) to detect a difference of 1 score in endoscopic scores. A difference of 1 was deemed to be clinically relevant as the endoscopic scoring ranges from 0 to 4 using increments of 1.

Bronchoalveolar lavage cytology was used in this study as it is recommended for the definitive diagnosis of mild to moderate asthma especially when mast cells are of importance as these cells are not typically represented in samples obtained via tracheal washes.^38^

In horses with severe asthma, BALF neutrophilia does not always normalize after effective treatment with systemic or inhaled corticosteroids, particularly when environmental conditions are not improved.^9^, ^39^, ^40^ In the study reported here, treatment with inhaled ciclesonide resulted in a significant decrease in mast cell percentage in the BALF of treated horses but not in the placebo group. Considering that the horses' routines and environments were not changed, this strongly suggested that the decrease in BALF mastocytosis was due to the inhaled ciclesonide therapy. While it is possible that the duration of treatment might have influenced the results, others have treated severe asthma for 10 days using inhaled corticosteroids with no changes in BALF neutrophilia.^40^ Severity and chronicity of disease might also explain the difference in cell response as many previous reports are related to severe asthma.^9^, ^39^, ^40^ The effect of ciclesonide, the type of cell and the particle size produced by this inhaler designed for horses might have also been responsible for the reduction in mast cells. Only 11 horses in this study had a neutrophilic inflammation, and 1 had an abnormal percentage of eosinophils in BALF. Thus, we were unable to draw conclusions related to the efficacy of ciclesonide in moderate asthma caused by either one of these cell types.

Reports of cytokine expression in the BALF of horses with mild to moderate asthma have been variable. Some studies report an upregulated expression of mostly Th2 cytokines^30^ whereas others reported a Th17 response^41^ or increased IL‐1b and IL‐10 in BALF.^42^ The type of cells present in the BALF of these horses and the environment where horses are housed might explain these discrepancies.^42^ In addition, the stage of disease and the timing of sample collection also affect cytokine expression in BALF.^43^ In our study, the relative expression of IL‐6, a proinflammatory cytokine that increases in the BALF of horses with moderate asthma,^30^ significantly decreased over time in treated horses. This was likely associated with the observed decrease in BALF mast cells in this group as IL‐6 promotes mast cell number and activities and plays an important role in the pathogenesis of mastocytic asthma in humans.^44^

IL‐13 is a proinflammatory cytokine produced by both, T and non‐T cells including mast cells.^45^ In our study, treatment with ciclesonide resulted in decrease expression of IL‐13 overtime that was not seen in the control group. This was expected given the decrease in BALF mast cell percentage after treatment and mirrors that seen after treatment of human mastocytic asthma with dexamethasone.^45^ In addition, corticosteroids appear to have a direct suppressive effect in the production of IL‐13 by mastocytes through an inhibitory action on the cell's gene expression.^45^

Environmental and management factors were unchanged during the study to decrease the potential effect of this factor on the results. Although the dust composition in the rebreathing zone of the horses was not measured, the research personnel was present on‐site daily during the study and did not record any changes in the environmental or management conditions. This met the goal of this study in assessing the validity of ciclesonide treatment under realistic field conditions.

Our study has limitations. The number of horses included in the study was relatively small, but the fact that they were all housed in the same facility, were of the same breed and had similar management, decreased variablity. Furthermore, all personnel and researchers involved in the study were blinded, significantly minimizing potential biases. While the study aimed to include horses with chronic cough, information related to the specific duration of the horses' clinical signs prior to enrollment in this study was not collected. The long‐term effect of the treatment was not evaluated.

In summary, a 10‐day treatment regimen with inhaled ciclesonide was well tolerated and significantly improved clinical signs and lower airway mastocytic inflammation of Thoroughbred racehorses with moderate asthma. The fact that improvement was observed without environmental changes at the racetrack and the short withdrawal time for racehorses makes ciclesonide a suitable treatment choice for moderate asthma when commercially available.

## CONFLICT OF INTEREST DECLARATION

Authors declare no conflict of interest.

## OFF‐LABEL ANTIMICROBIAL DECLARATION

Authors declare no off‐label use of antimicrobials.

## INSTITUTIONAL ANIMAL CARE AND USE COMMITTEE (IACUC) OR OTHER APPROVAL DECLARATION

Approved by the Animal Use and Care Committee at Washington State University. Horses were included only after owner or trainer consent was obtained.

## HUMAN ETHICS APPROVAL DECLARATION

Authors declare human ethics approval was not needed for this study.
